# Syphilis and neurosyphilis: HIV-coinfection and value of diagnostic parameters in cerebrospinal fluid

**DOI:** 10.1186/s40001-015-0175-8

**Published:** 2015-10-07

**Authors:** V. Merins, K. Hahn

**Affiliations:** Group Practice Family Physicians, Alt-Buckow 9-11, 12349 Berlin, Germany; Department of Neurology, Universitätsmedizin Charité, Charitéplatz 1, 10117 Berlin, Germany

**Keywords:** Syphilis, Neurosyphilis, Cerebrospinal fluid, HIV

## Abstract

**Background:**

Neurosyphilis might be difficult to diagnose particularly in asymptomatic patients and patients with HIV-coinfection. The objective of this study was to evaluate current diagnostic standards for neurosyphilis in HIV-positive and -negative patients.

**Methods:**

We studied retrospectively patients with an active syphilis infection who had additionally undergone lumbar puncture. Patients where the criteria for the diagnosis of a definite or probable neurosyphilis were applicable were further analyzed for clinical symptoms, CSF, HIV-status as well as *Treponema pallidum* testing in serum and CSF. Correlation analysis of categorical variables was done by using the Chi-square test or in cases of small sample sizes the exact test of Fisher. *p* values ≤0.05 were considered significant.

**Results:**

Eighty-nine patients were diagnosed with syphilis. All necessary criteria for the diagnosis of a neurosyphilis were available in 67 of them including 35 HIV-positive and 32 HIV-negative patients. A definite neurosyphilis could be retrospectively diagnosed in 13 and a probable in another 25 cases. Normal CSF results were more likely in HIV-negatives (*p* = 0.016). A neurosyphilis was correlated to a CSF pleocytosis > 5 cells/µl and to an albumin quotient >7.8 mg/dl regardless of a parallel HIV infection. HIV-positives had more frequently a CSF-RPR titre >1:4 than HIV-negatives (*p* = 0.031). However, the RPR test in CSF in definite or probable neurosyphilis had a sensitivity of only 21 %.

**Discussion:**

Our data show that a pleocytosis and an elevated albumin quotient correlate with neurosyphilis. However, the CSF-RPR test as gold standard in neurosyphilis diagnostics has a very low sensitivity.

## Background

The incidence of syphilis rises especially in patients with a high risk for HIV [1–6]. Neurosyphilis might be difficult to diagnose particularly in asymptomatic patients and patients with HIV coinfection. It was shown that the HI-viral load during a syphilis infection is higher than without coinfection and moreover, it decreases when syphilis is treated [7]. Additionally even without a syphilis infection, HIV-infected patients often show a CSF pleocytosis and an increased protein level [8]. The importance of considering HIV-coinfection as an important factor for the development of neurosyphilis was displayed in a study by Lynn and Lightman in 2004. They detected that 23.5 % of untreated patients with HIV-coinfection had a neurosyphilis while this applied to only 10 % of untreated patients without HIV [9].

According to the German guidelines, the diagnosis of neurosyphilis is based on clinical symptoms and examination of the cerebrospinal fluid (CSF) for inflammatory parameters and treponemal and non-treponemal tests like TPPA-, RPR-, and FTA-ABS test. However, a pleocytosis and elevated CSF-protein can indicate neurosyphilis although it is difficult to discriminate between alterations due to HIV-infection or Neurosyphilis. Another parameter indicating neurosyphilis is a positive CSF-TPPA- or -FTA-ABS test. The activity of neurosyphilis can be confirmed by a positive CSF-VDRL or CSF-RPR although it is important to be aware of the tests sensitivity which ranges between 27 and 70 % [8, 10, 11].

Another parameter which is included in the German guidelines for “sexually transmitted diseases for diagnosis and therapy in syphilis” to diagnose neurosyphilis is the TPPA- or ITPA index. It is calculated by using the TPPA-titre in relation to the IgG-values in serum and CSF. Its sensitivity is higher than the sensitivity of the CSF-VDRL but it is not suitable as a parameter for activity [8, 10–12]. Only a positive CSF-VDRL- or -RPR-test result is suitable to confirm activity as well as specific IgM-antibodies in CSF or a pleocytosis. In addition to that a blood-CSF barrier disturbance should be taken into account [12].

Regarding this it would be useful to find predictive parameters for neurosyphilis. Lately a number of CD4+ T cells <350/μl and a serum RPR-titre >1:32 were interpreted as predictive [13–15].

The objective of this study was to evaluate current diagnostic standards for their sensitivity and specificity in serum and cerebrospinal fluid (CSF) in HIV-positive and -negative patients with active syphilis and to evaluate predictive factors for neurosyphilis.

## Methods

We screened electronic medical files of the Charité Hospital from 2000 to 2012 for patients with a confirmed active syphilis infection who had undergone lumbar puncture.

All patients had a positive TPPA-titre (≥1:80) and positive FTA-ABS OR IgM-FTA-ABS in serum. Patients where the criteria for the diagnosis of a definite or probable neurosyphilis were applicable were further analyzed.

We searched the medical records for HIV-status and for clinical symptoms such as focal deficits (i.e. dysarthria/aphasia, vertigo, cognitive impairment, ocular involvement, gait disturbance, hemiplegia, incontinence, and cranial nerve palsy), affective signs (i.e. depression), pain (i.e. neuropathic pain, headache), fatigue, quantitative disturbance of consciousness and seizures.

We analyzed the CSF for cell count, albumin quotient, protein content, glucose, lactate, oligoclonal bands and quantitative IgG, IgM and IgA and considered a CSF protein level >50 mg/dl OR albumin quotient >7.8 as a blood-CSF barrier disturbance. Furthermore we built subpopulations regarding the inflammatory response [1: inflammatory CSF (pleocytosis with/without blood-CSF barrier disturbance), 2: pure blood-CSF barrier disturbance, 3: intrathecal immunoglobulin synthesis, 4: normal CSF].

We also analyzed the CSF and serum for *Treponema pallidum* specific tests by using *Treponema pallidum* particle agglutination test (Serodia^®^ TP·PA), Rapid Plasma Reagin-test (RPR-nosticon^®^ II, BioMérieux) and IgM-ELISA or 19S-IgM-FTA-ABS test and also the ITPA index.

Criteria of a neurosyphilis were retrospectively applied to the patients. According to the Guideline of the German Sexually Transmitted Diseases Society for diagnosis and therapy of syphilis we used the following criteria to diagnose a definite neurosyphilis [12]: ITPA index >2 AND positive CSF-IgM-FTA ABS OR CSF-RPR-titre >1:1 OR an inflammatory CSF syndrome (pleocytosis >4 cells/µl OR blood-CSF barrier disturbance).

Neurosyphilis was probable if two of the first three following conditions were fulfilled and in addition to that the 4th condition always had to be fulfilled [12]:

1. Chronically progressive course of neurologic-psychiatric symptoms with phases of aggravation and partly remission.
2. Pathological CSF with mixed cellular or mononuclear pleocytosis (>4 cells/μl), blood-CSF barrier disturbance (CSF-protein >0.5 g/l or albumin quotient >7.8) and/or IgG-dominant immune response in central nervous system.
3. Beneficial effect of antibiotics on the course of the disease and/or pathological CSF (particularly pleocytosis and barrier disturbance).
4. Positive TPHA (or TPPA) and FTA-abs in serum.

The 4th condition applied to all of our patients since it was a criterion for inclusion in this study. The 3rd condition cannot be evaluated retrospectively because the course of the disease was not observed. Patients who met the first two criteria were regarded as patients with probable neurosyphilis. We did not use the diagnosis of neurosyphilis by a clinician as a parameter.

The ethics committee of the Charité School of Medicine in Berlin approved the study. The general terms of data protection and the Charité ‘Good Medical and Scientific Practice’ statutes were applied.

### Statistics

We used SPSS 18^®^ for statistical analysis. Scaled variables were tested by Shapiro–Wilk-test for normal distribution. The Mann–Whitney *U* test for independent samples was used to compare these variables in the group of HIV-positive and HIV-negative patients. Correlation analysis of categorical variables was done by using the Chi-squared test or in cases of small sample size the exact test of Fisher. Multivariate analysis was done by binary logistic regression analysis. *p* values ≤0.05 were considered significant.

## Results

We found 89 patients who were diagnosed with active syphilis and underwent lumbar puncture at Charité Berlin between 2000 and 2012. These included 80 male and 9 female patients. Retrospectively we were able to get information about the HIV-status for 75 of these individuals including 39 HIV-positive and 36 HIV-negative patients. The following results are based on the analysis of these according to the available criteria to diagnose either definite or probable neurosyphilis.

Furthermore all necessary criteria for the diagnosis of a definite or probable neurosyphilis were available in 67 of them including 35 HIV-positive and 32 HIV-negative patients. Criteria for a definite neurosyphilis could be retrospectively fulfilled by 13 cases with six HIV-positive and six HIV-negative men and one HIV-negative woman. Criteria for a probable neurosyphilis were fulfilled by another 25 cases including one HIV-negative woman and 24 men, of whom 17 are HIV-positive and 7 HIV-negative (Table 1).

**Table 1:** *Treponema pallidum* testing and CSF characteristics in individuals with a retrospectively definite or probable neurosyphilis divided according to HIV status

	Neurosyphilis+				Neurosyphilis−			
	*N* = 38				*N* = 29			
	HIV+		HIV−		HIV+		HIV−	
	*N* = 23		*N* = 15		*N* = 12		*N* = 17	
		*n*		*n*		*n*		*n*
CSF cell count in cells/µl (mean/range)	87 (1–511)	23	32 (4–165)	15	9 (0–33)	11	9 (1–50)	16
					n.a.	1	n.a.	1
CSF Protein in mg/dl (mean/range)	129 (40–928)	23	86 (14–437)	15	145 (23–650)	11	43 (25–60)	16
						1	n.a.	1
Albumin quotient in (mean/range)	12 (3.2–31.4)	20	8.4 (2.7–15.9)	14	6.1 (3.1–15.7)	11	6.4 (3.6–10.7)	14
	n.a.	3	n.a.	1	n.a.	1	n.a.	3
Oligoclonal bands in CSF	pos.	11	pos.	8	pos.	9	pos.	6
	neg.	8	neg.	5	neg.	2	neg.	8
	n.a.	4	n.a.	2	n.a.	1	n.a.	3
Serum RPR (median/range)	1:32 (1:1–1:1024)	21	1:16 (1:2–1:512)	13	1:32 (1:4–1:128)	11	1:16 (1:2–1:256)	14
		2 neg.		2 neg.		1 neg.		2 neg.
CSF RPR (median/range)	1:8 (1:8–1:512)	5	1:2/1:4 (1:1–1:4)	4	neg.	12	neg.	16
	neg.	18	neg.	11			pos.	1
ITPA index (mean/range)	4.1 (0–30.9)	21	5 (0.1–15.2)	13	0.9 (0–5.8)	12	1 (0.1–3.7)	16
	n.a.	2	n.a.	2			n.a.	1
CD4 cell count in cells/µl (mean/range)	333 (60–780)	18	–	–	320 (41–611)	10	–	–
	n.a.	5			n.a.	2		

*neg.* negative, *pos.* positive, *n.a.* not available

### Differences between HIV-positives and HIV-negatives

Thirteen patients fulfilled the criteria of a definite neurosyphilis. Half of the HIV-positive patients (3/6) have been asymptomatic for neurological symptoms while the HIV-negatives have been symptomatic in all cases (7/7).

Further we found no difference in neurological clinical symptoms between HIV-positive and HIV-negative patients with either definite or probable neurosyphilis (*p* = 0.264). However the clinical spectrum had a very wide range and the subpopulations were too small to reach statistical significance.

#### Cerebrospinal fluid

The protein level overall was higher in HIV-positives (*p* = 0.002) with a mean of 128.4 mg/dl (23–928 mg/dl) in comparison with HIV-negatives with a mean of 71.5 mg/dl (14–437 mg/dl). Dividing into subgroups with or without neurosyphilis, the difference between HIV-negatives and -positives becomes hardly insignificant. Without neurosyphilis the mean protein level in HIV-positives was 126.8 mg/dl (23–650 mg/dl) and 60.6 mg/dl (25–389 mg/dl) in HIV-negatives (*p* = 0.064). With definite neurosyphilis the mean protein level was 115.5 mg/dl (44–194 mg/dl) in HIV-positives and 57.7 mg/dl (14–149 mg/dl) in HIV-negatives (*p* = 0.063).

The mean cell count in CSF was 60 cells/µl (0–511 cells/µl) in HIV-positives and 19 cells/µl (1–165 cells/µl) in HIV-negatives. We found a mean albumin quotient of 9.5 mg/dl (3.1–31.4 mg/dl) in HIV-positive patients and a mean albumin quotient of 7.2 (2.7–15.9 mg/dl) in HIV-negatives.

We identified a total of 50 patients with an inflammatory CSF (group 1). Among these we found 28 patients with HIV and 22 HIV-negative patients. HIV-positives and -negatives did not differ in the frequency of occurrence of an inflammatory CSF (*p* = 0.226).

Patients with pure blood-CSF barrier disturbance without any other abnormalities in CSF were included in the second group. This applied to four patients including exclusively HIV-positive patients. Nevertheless, there is no significant difference between HIV-positives and HIV-negatives, which we assume is due to the small sample size (*p* = 0.076).

An isolated immunoglobulin synthesis with oligoclonal bands or quantitative synthesis of IgG and/or IgM in CSF (group 3) was found in eight patients. Among these were five HIV-positive patients and three HIV-negative patients. Again, the difference between the two subpopulations was insignificant (*p* = 0.420).

A normal CSF (group 4) was found in seven patients, all of whom were HIV-negative. HIV-negative patients had significantly more often a normal CSF than HIV-positive patients (*p* = 0.003).

### Subgroups with good or poor immune status defined by CD4+ cell counts

Furthermore we divided the HIV-positive patients with available CD4+ cell counts (*n* = 32) in those with a good immune status defined by CD4+cells above 350/µl and those with a significantly impaired immune status defined by a CD4+ cell count ≤350/µl.

We found 16 patients with good immune status with a mean CD4+ cell count of 546 cells/µl (357–810 cells/µl) and 16 patients with poor immune status with a mean of 167 cells/µl (41–288 cells/µl).

28 Of those 32 HIV-positive patients could be analyzed according to the necessary data to diagnose either definite or probable neurosyphilis. We discovered a significant difference in the occurrence of headache (*p* = 0.022) and neurological symptoms (*p* = 0.037) which were both more likely in patients with CD4+ cell count ≤350/µl. There was no difference in diagnostic parameters to be found.

### Predictive factors for neurosyphilis

Another objective was to evaluate predictive factors for neurosyphilis. We therefore analyzed only those 28 patients with available CD4+ cell count and all necessary criteria for the diagnosis of definite or probable neurosyphilis. We calculated the positive predictive value of the serum RPR-titre ≥1:32 as well as of the CD4+ cell count for a definite or probable neurosyphilis. The first reached a positive predictive value of 71.4 % and the second had a value of 66.7 %. The calculated positive predictive value of those two parameters in combination was 66.7 %, however the sensitivity of both parameters in combination increased to 77.8 % in comparison to considering both separately (55.6 % for both parameters). Considering this we cannot give the advice to use a poor CD4+ cell count or a high RPR-titre to predict a neurosyphilis (Table 2).

**Table 2:** Predictive parameters for definite or probable neurosyphilis

Parameter	Odds ratio	*p* value*
Serum-RPR-titre ≥32	1.53	0.383
CD4+ cell count ≤350/µl	1.1	0.612
Blood-CSF barrier disturbance in HIV-negatives	9	0.018**
Blood-CSF barrier disturbance in HIV-positives	6	0.027**
Albumin quotient >7.8 mg/dl in HIV-negatives	8.0	0.026**
Albumin quotient >7.8 mg/dl in HIV-positives	23.3	0.006**
Pleocytosis >5 cells/µl in HIV-negatives	6.7	0.006**
Pleocytosis >5 cells/µl in HIV-positives	8.3	0.015**
ITPA index >2 in HIV-negatives	7.6	0.025**
positive IgM-FTA-ABS test in HIV-negatives	13.1	0.022**

Predictive parameters and their *p* values and odds ratios concerning neurosyphilis

* p values are calculated with binary logistic regression

** Coefficient significant (5 %)

We tried to find other parameters in serum to predict neurosyphilis by using regression analysis for TPPA test, RPR test, IgM-FTA-ABS test, CD4+ cell count, HI-viral-load in serum and HIV-status. None of these showed a significant correlation with definite or probable neurosyphilis. Our data shows rather how important it is to look for more suitable parameters to predict a neurosyphilis.

We therefore evaluated parameters in CSF which might correlate with definite or probable neurosyphilis in 67 patients with known HIV-status who had all necessary criteria for the diagnosis of definite or probable neurosyphilis available. We found blood-CSF barrier disturbance (*p* = 0.001), albumin quotient >7.8 mg/dl (*p* = 0.0002), CSF pleocytosis >5 cells/µl (*p* = 0.0002), local IgG-synthesis (*p* = 0.047), CSF-TPPA ≥1:32 (*p* = 0.007), ITPA index >2 (*p* = 0.042) and positive IgM-FTA-ABS in CSF (*p* = 0.016) to be correlated. Those patients had significantly more often a definite or probable neurosyphilis.

A disturbed blood-CSF barrier significantly correlates with a definite or probable neurosyphilis for HIV-positives (*p* = 0.027, odds ratio 6.0) as well as for HIV-negatives (*p* = 0.018, odds ratio 9.0). The same applies for an albumin quotient >7.8 mg/dl with *p* = 0.006 in HIV-positives (odds ratio 23.3) and *p* = 0.026 in HIV-negatives (odds ratio 8.0). Another parameter in CSF correlating with definite or probable neurosyphilis in both subgroups was a CSF pleocytosis >5 cells/µl (*p* = 0.015 and odds ratio 7.3 in HIV-negatives and *p* = 0.006 and odds ratio 9.5 in HIV-positives). A CSF-TPPA ≥1:32 significantly correlates with definite or probable neurosyphilis only in the subgroup of HIV-positives (*p* = 0.003) but no longer for HIV-negatives. In contrast to this an ITPA index >2 correlates significantly with definite or probable neurosyphilis only in the subgroup of HIV-negatives (*p* = 0.025, odds ratio 8.2). The same applies to a positive IgM-FTA-ABS in CSF with *p* = 0.022 and odds ratio 14.0 for HIV-negatives.

Considering the subgroups, there was no longer a correlation detectable for local IgG-synthesis.

### Sensitivity and specificity of *Treponema pallidum* testing in cerebrospinal fluid

#### CSF-RPR test

The RPR test in CSF plays an important role in the diagnosis of neurosyphilis and is the gold standard for diagnosis of neurosyphilis next to the VDRL test in CSF. In our analyzed group of patients with available HIV-status and all necessary criteria for the diagnosis of definite or probable neurosyphilis (*n* = 67), the RPR test in CSF has a sensitivity of only 21 % and a specificity of 97 % for a definite or probable neurosyphilis. Accordingly almost 80 % of neurosyphilis cases would have been overlooked using this test for the diagnosis of neurosyphilis.

The sensitivity of the CSF-RPR based on definite neurosyphilis increases to 31 %. However, this is still very low because more than two-thirds of patients with definite neurosyphilis would have been overlooked by this test.

If we separate patients with a definite and probable neurosyphilis by their HIV-status the sensitivity of the RPR test in CSF would be 22 % in HIV-positive patients and 20 % in HIV-negatives. Concerning definite neurosyphilis the sensitivity of the RPR test in CSF increases in HIV-coinfected patients to 33 % and in HIV-negative to 29 %.

#### CSF-FTA-ABS test

The CSF-FTA-ABS is often used to exclude the diagnosis of neurosyphilis, since its sensitivity in contrast to CSF-RPR or CSF-VDRL is approximately 100 %. The specificity has been described with just over 70 % [13, 16].

In our analysis the CSF-FTA-abs showed a sensitivity of 89 % and a specificity of 22 %. The criteria used in this study to diagnose a definite or probable neurosyphilis included clinical symptoms. This test would have failed to see three cases we classified as definite or probable neurosyphilis. Concerning definite neurosyphilis, which is based more on laboratory diagnostic criteria, the sensitivity of the CSF-FTA-abs was 90 % with a specificity of 18 %. According to this the CSF-FTA-abs would have excluded the diagnosis of neurosyphilis in one patient who met our criteria for definite neurosyphilis.

Differentiating the specificity and sensitivity according to HIV-status, the CSF-FTA-ABS has a specificity of 44 % and a sensitivity of 83 % in the diagnosis of definite or probable neurosyphilis in the group of HIV-coinfected patients. In the group of HIV-negative patients, the sensitivity is 100 % and the specificity only 7 %.

#### ITPA index

In our study we observed a correlation between an ITPA index >2 and neurosyphilis (*p* = 0.042). However, if we discriminate between HIV-positives and -negatives this correlation is only detectable in HIV-negative patients (*p* = 0.025) and not in HIV-positives (*p* = 0.448). The sensitivity was 54 % and the specificity was 88 % for the ITPA index >2 in HIV-negatives for neurosyphilis.

## Discussion

We have retrospectively evaluated current diagnostic standards for neurosyphilis in HIV-positive and -negative patients in patients who were previously diagnosed with active syphilis and who had undergone lumbar puncture.

### Sensitivity and specificity of cerebrospinal fluid analysis

The CSF-RPR had a sensitivity of 21 % and a specificity of 97 % in the diagnosis of probable or definite neurosyphilis. This corresponds approximately to the sensitivity and specificity of the CSF-VDRL and thus to the results of Castro et al. [10, 11, 17]. We were not able to determine the sensitivity and specificity of the CSF-VDRL in this study because in our population it was the CSF-RPR being used instead of the CSF-VDRL. However, a comparison between the RPR test used herein and reported in the literature with the sensitivity and specificity of CSF VDRL test is possible. If the CSF is not visibly contaminated with blood, a positive CSF-VDRL is for 100 % specific with a sensitivity of 27–70 % [10, 11]. The CSF-RPR indicated in one study the same sensitivity and specificity as the CSF-VDRL, thus both tests are applicable to diagnose neurosyphilis [17]. In this study none of the samples was visibly contaminated with blood.

CSF-RPR is used for the diagnosis of neurosyphilis, but its sensitivity is very low. This raises the question whether a test with such a low sensitivity actually should continue to be used as a gold standard for neurosyphilis diagnosis, if it overlooks almost 80 % of neurosyphilis patients.

Taking the other available tests into account the diagnosis of neurosyphilis still remains difficult. The CSF-FTA-abs had in some studies a sensitivity of 100 %, but it is not as specific as the CSF-VDRL or -RPR [16, 18]. Thus this test seems to be suitable to exclude neurosyphilis. However, these statements are based on common laboratory diagnostic definitions of neurosyphilis and may not be suitable to exclude neurosyphilis when strong clinical suspicion exists [19, 20]. Our data analysis raises the suspicion that some cases (3/28, 11 %) of definite or probable neurosyphilis would be incorrectly excluded since the CSF-FTA-ABS had a sensitivity of 89 % in the diagnosis of neurosyphilis. This included one case with definite and two cases with probable neurosyphilis.

A positive CSF TPPA test, particularly if it is confirmed by an activity parameter, may help to diagnose neurosyphilis. This is also suggested by the observation that using the German guidelines three potential neurologically asymptomatic neurosyphilis patients would have been overlooked yet all of them had a positive CSF TPPA.

In addition, the ITPA index (sensitivity 84 % specificity, 100 %) is used in Germany [12]. The ITPA index seems to be only usefully applicable in the diagnosis of neurosyphilis in HIV-negative patients since there was no correlation in HIV-positive patients between neurosyphilis and positive ITPA index.

Unfortunately activity parameters for the confirmation of neurosyphilis which have a sufficiently high sensitivity are lacking. As described above, only the CSF-VDRL, CSF-RPR as well as a specific IgM antibody result or pleocytosis would be suitable to accomplish this task. The VDRL and RPR test have such a low sensitivity that they would confirm the activity of neurosyphilis in only a few cases. A pleocytosis may be used uncritically only in HIV-negative patients since HIV-positives can show pleocytosis without having any other infection.

As already described by other authors an increased CSF protein concentration can occur in HIV-positive patients without neurosyphilis [21]. In our study the mean protein concentration in HIV-positives in total was even 128 mg/dl, which is much higher than the limit of 40 or 50 mg/dl which is used in the guidelines of the German Society for STD diagnosis and treatment of syphilis to identify a probable neurosyphilis [12]. An elevated protein concentration in CSF is thus not suitable as a parameter for confirmation of neurosyphilis in HIV-positives.

However a disturbed blood-CSF barrier significantly correlates with a definite or probable neurosyphilis for HIV-positives (*p* = 0.027) as well as for HIV-negatives (*p* = 0.018). The same applies for an albumin quotient >7.8 mg/dl with *p* = 0.006 in HIV-positives and *p* = 0.026 in HIV-negatives. These two parameters should be given greater consideration for the diagnosis of neurosyphilis.

### Clinical manifestation of syphilis in HIV-positives and -negatives

In our cohort HIV-coinfected patients had more often a primary lesion than HIV-negatives. Unfortunately we cannot evaluate the frequent occurrence of multiple chancres and their persistence in HIV-positives retrospectively which was described in literature [22–24]. Although we found no difference between HIV-coinfected patients and HIV-negatives concerning the spectrum of neurological symptoms, we detected that HIV-negatives always presented with neurological symptoms, while HIV-positives have been also asymptomatic. Altogether 8.6 % of the HIV-positives had a neurologically asymptomatic neurosyphilis which matches with the results of Holtom et al. who found 9 % of the HIV-positive patients to have an asymptomatic neurosyphilis [25].

All in all our results show that clinical symptoms do not differ as much between HIV-positives and -negatives as we would have expected. But still the higher incidence of neurologically asymptomatic neurosyphilis during HIV-coinfection shows that these patients are at risk to miss a lumbar puncture to provide the right diagnosis and to undergo the necessary therapy.

Neuroimaging is also very important as a diagnostic parameter for neurosyphilis [26]. Unfortunately we were not able to analyse imaging data since two-thirds of the patients in our study have not received neuroimaging.

### Neurosyphilis, HIV immune status and predictive parameters

We tried to ascertain whether a poor or good immune status in HIV-coinfected patients, which we defined by a number of CD4+ T cells < or ≥350/μl, increases the risk for neurosyphilis or not. Our results show no difference in these two groups concerning the diagnostic parameters but we found out that patients with poor immune status had a higher incidence of probable neurosyphilis while patients with better immune status were more likely to have a neurologically asymptomatic neurosyphilis. Unfortunately a good or poor immune status does not help to predict or exclude the diagnosis of neurosyphilis. It is rather apparent that HIV-coinfected patients are generally more likely to have neurosyphilis and maybe they are even more likely to have an asymptomatically proceeding disease which should not be missed by the physician. The role of a good or poor immune status should be explored in further studies.

Furthermore we found neither a correlation between immune status and neurosyphilis nor between RPR-titre and neurosyphilis. The combination of both parameters also only had a predictive value of 66.7 %. This matches with the study of Wang et al. who showed that patients who did not meet the criteria of poor CD4+ T cells or RPR-titre ≥1:32 still had neurosyphilis [27]. We admit that the difference between our results and those suggesting that poor CD4+ T cells or high serum-RPR-titre are predictive for neurosyphilis might be due to the fact that we used other criteria for the definition of neurosyphilis.

Altogether our results show that more research concerning diagnostic parameters in serum and cerebrospinal fluid is necessary.

## Conclusions

This study indicates that neurosyphilis is still difficult to diagnose particularly in asymptomatic patients and patients with HIV-coinfection. A spinal tap should be an essential part of the diagnostic workup in any patient especially in those with HIV coinfection. Our data show that a pleocytosis and an elevated albumin quotient correlate with neurosyphilis. However, standard diagnostic tests such as the CSF-RPR have a sensitivity that is far too low to be reliable in clinical settings.
